# On geometric mean fitness: a reply to Takacs and Bourrat

**DOI:** 10.1007/s10539-022-09874-x

**Published:** 2022-08-26

**Authors:** Bengt Autzen, Samir Okasha

**Affiliations:** 1 University College Cork, Cork, Ireland; 2 University of Bristol, Bristol, UK

**Keywords:** Evolution, Fitness, Geometric mean, Population growth, Stochasticity

## Abstract

In a recent paper, Takacs and Bourrat (Biol Philos 37:12, 2022) examine the use of geometric mean reproductive output as a measure of biological fitness. We welcome Takacs and Bourrat’s scrutiny of a fitness definition that some philosophers have adopted uncritically. We also welcome Takacs and Bourrat’s attempt to marry the philosophical literature on fitness with the biological literature on mathematical measures of fitness. However, some of the main claims made by Takacs and Bourrat are not correct, while others are correct but not for the reasons they give.

## A false contrast

The concept of geometric mean fitness arose from attempts to understand how natural selection works in a fluctuating (or stochastically-varying) environment (Lewontin and Cohen 1969; Gillespie 1973). The simplest such scenario involves a large population of haploid asexual organisms, with discrete non-overlapping generations and no density regulation. Organisms are of two (heritable) types. An organism’s reproductive output depends on both type and environmental state. If the environmental state varies stochastically from generation to generation, then in the long-run, the type with the highest value of $G(X) = \prod _{i} X(i)^{p(i)}$ will come to dominate the population, where *X*(*i*) is the per-capita number of surviving offspring of type *X* in environmental state *i*, *p*(*i*) is the probability that state *i* occurs, and the product is taken over environmental states. Note that the geometric mean can equivalently be written as $G(X) = \exp {(\sum _{i} ln X(i)\cdot p(i))}$.

Takacs and Bourrat observe that a number of philosophers have advocated geometric mean number of offspring as “the” definition of biological fitness, often without reference to the underlying model assumptions (and some have tried to extract general philosophical morals from this) (Beatty and Finsen 1989; Brandon 1990; Sober 2001). Takacs and Bourrat are right to query this tendency. However, they go on to contrast geometric mean fitness with the instantaneous rate of natural increase *r* (or Malthusian parameter), which is defined as the exponential population growth rate on a continuous time scale. Takacs and Bourrat then assert that geometric mean fitness amounts to a special case of the Malthusian parameter *r* in the case of a discrete population model with non-overlapping generations. They write:

Fitness construed as a continuous growth rate is a more general measure. [...] However, fitness is not measured by way of the geometric mean when continuous growth rates are used (2022, 17)

This assessment can lead to some mistaken ideas about the relationship between geometric mean fitness and population growth. It is true that the geometric mean principle presupposes discrete time and non-overlapping generations (as does much of classical population genetics). And it is also true that these idealizations are not appropriate for many species (as has often been observed). So, Takacs and Bourrat are right that “defining” fitness as geometric mean number of offspring rests on model assumptions. However, in continuous time models, which permit overlapping generations, there is in fact a quantity that is strictly analogous to the geometric mean fitness, known as the *long-run growth rate*, which determines the evolutionary outcome in a fluctuating environment (Lande 2007; Saether and Engen 2015). So the concept of maximizing geometric mean fitness generalizes easily to the continuous setting, contrary to what Takacs and Bourrat imply.

The long-run growth rate, usually denoted *s*, is defined as the asymptotic rate of increase of the natural logarithm of population size. To illustrate the relationship between geometric mean fitness and long-run growth rate, consider a population consisting of $N_{t}$ identical individuals of the same haploid genotype at time *t* ($t \in \{0,1,2,...\}$) that grows without density regulation.^1^ Generations are non-overlapping, and the environmental variation affecting population growth is assumed to be stochastically independent over time and follows the same probability distribution. The population size at time *t* is given by $N_{t}=N_{0}W_{0}W_{1}...W_{t-1}$ with the growth multipliers $W_{t}$ (frequently referred to as “fitness” in the biological literature). The logarithmic population size $ln N_{t}$ can then be expressed as $ln N_{t} = ln N_{0} + \sum _{u=1}^{t}ln W_{u}$. The slope of the logarithmic population size $ln N_{t}$ is given by $\frac{1}{t} (ln N_{t}- ln N_{0})$, which reduces to $\frac{1}{t} \sum _{u=1}^{t} ln W_{u}$, which, by the law of large numbers, converges to *E*(*lnW*) as *t* goes to infinity. (Since the $W_{t}$ are independent and identically distributed (i.i.d.), we can use the shorthand *W* to write *E*(*lnW*), i.e. the expectation, over the uncertain environment, of the natural logarithm of *W*.) Thus over long time intervals the population grows exponentially with growth rate $s =E (ln W)$.

In this discrete-time setting, maximization of the long-run growth rate is identical to maximization of geometric mean fitness. For the geometric mean of a strictly positive random variable *X* is given by $\exp {(E (ln X))}$. Since the long-run growth rate *s* is equal to *E*(*lnW*), as we have seen, the geometric mean fitness is simply $e^{s}$. Therefore, choosing a genotype based on the long-run growth criterion (maximal *s*) simply amounts to applying the geometric mean principle. However, the long-run growth rate measure applies more widely, in particular to continuous-time models that allow for overlapping generations (Tuljapurkar 1982; Lande et al. 2003).

The expected value *E*(*W*), referred to as the finite rate of increase in the biological literature and denoted by $\lambda$, does not determine the growth of the genotype over a long period of time in a stochastic environment. Since the instantaneous rate of natural increase *r* is given by $e^{r} = \lambda$, Jensen’s inequality tells us that $s \le r$ holds (Starrfelt and Kokko 2012). It can be shown that in large populations where demographic stochasticity can be ignored, the long-run growth rate *s* is approximately equal to $r- \frac{\sigma ^{2} }{2 \lambda }$, with $\sigma ^{2}$ denoting the variance for the temporal fluctuations in *W* (Lewontin and Cohen 1969; Tuljapurkar 1982). That is, in these models the long-term growth rate *s* differs from the instantaneous rate of natural increase *r* by a correction factor capturing the environmental stochasticity.

This shows that Takacs and Bourrat are quite wrong to say that the geometric mean fitness is a special case of the instantaneous rate of natural increase. With a fluctuating environment, it is not true that the type with the highest Malthusian parameter $r=ln E(W)$ will dominate the population. What is true is something very different, namely that geometric mean fitness is a special case of the long-run growth rate. This shows, further, that Takacs and Bourrat’s emphasis on the distinction between discrete time models with non-overlapping generations and continuous time models with overlapping generations is misplaced. These two are simply alternative modeling choices. The key biological implication of the early work on geometric mean fitness—that natural selection will favour genotypes with lower variance in per-capita reproductive output, other things being equal—applies equally in continuous-time models where the environment varies stochastically.

## A cautionary tale

The subtitle of Takacs and Bourrat’s paper—“a cautionary tale about the use of the geometric mean as a measure of fitness”—leads the reader to expect that they will identify circumstances in which this fitness measure is inappropriate (or leads to the wrong prediction). However, they do not do this. Rather, they make two points: (i) since the geometric mean of a random variable *X* is inter-definable with the arithmetic mean of *lnX*, the arithmetic mean is “on an equal footing” with the geometric mean, so remains a “good measure of fitness” (p.12); (ii) the geometric mean is appropriate for models with discrete time and non-overlapping generations only (p.12). Neither (i) nor (ii) is a well-taken objection. Point (i) is obviously true, but shows only that we need to take care to specify what random variable we are talking about, when we ask whether the geometric or arithmetic mean of that variable is a “better” measure. Point (ii) is true but beside the point, given that the long-run growth rate is the analogue for continuous-time models, as noted above.

That being said, in the spirit of Takacs and Bourrat’s “cautionary tale”, some cautionary remarks are indeed in order. There are real reasons why the geometric mean fitness (or long-run growth rate) criterion may be questionable. We will briefly discuss two. The first is that stochastic models in which geometric mean fitness offers the appropriate criterion of evolutionary success rely on the assumption of a sufficiently large population. For instance, Gillespie’s demonstration that geometric mean fitness is maximized under environmental stochasticity assumes that the competing strategies grow without density or frequency-dependence and thus that the population size can grow to infinity (Gillespie 1973); this means that we can make a certain prediction about what will eventually happen. As shown by Proulx and Day (2001), however, the fixation probability of a rare allele sometimes offers a better way of predicting evolutionary success in a finite population. Proulx and Day examine a model of lottery competition in a stochastic environment. In this model, sessile marine organisms, such as coral reef fish, compete for a small number of sites and are chosen randomly to win these sites. The population size is limited due to the finite number of available sites. Environmental stochasticity is introduced via an environmental variable affecting the mortality of adult organisms. Proulx and Day show that for small population sizes the fixation probability predicts evolutionary success, but as the population size becomes large the predictions based on geometric mean fitness turn out to be correct.

Secondly, the use of geometric mean fitness has been criticised for its emphasis on predicting the eventual fate of an allele (or genotype) while saying nothing about short-term evolutionary dynamics. Lande (2007) presents an example of two genotypes facing weak selection. Although the type with the higher long-run growth rate will ultimately dominate the population (i.e., its frequency approaches one), the superior type faces a long period of time where there is a significant probability that its frequency is close to zero. Lande’s example demonstrates that it can be unclear which fitness measure is most suitable for predicting evolutionary outcomes over finite time periods. Model assumptions and the context of inquiry—i.e. what evolutionary question we want to answer— jointly determine whether a candidate fitness measure is adequate. So there is indeed a case for “caution” when it comes to the use of geometric mean fitness, however for reasons entirely different from the ones that Takacs and Bourrat give.

In a similar vein, caution is needed when using the instantaneous rate of natural increase *r* to predict evolutionary success, as championed by Takacs and Bourrat. In a constant environment, *r* is often an appropriate measure of a genotype’s fitness—but not always. In an important article, Mylius and Diekmann (1995) contrast two possible fitness measures in the context of life-history evolution in a density-regulated population. The first is *r*; while the second is $R_{0}$, the net reproductive rate, defined as a female’s lifetime expected number of offspring. A key difference between these measures is that $R_{0}$ simply counts the total amount of offspring a female produces, so is insensitive to early versus late reproduction, unlike *r*.^2^ Using ESS (evolutionary stable strategy) considerations, Mylius and Diekmann show that whether *r* or $R_{0}$ is the “right” definition of fitness (in the sense of supplying a criterion for whether a genotype will be uninvasible by mutants once fixed in the population), depends on the precise form that the density regulation takes (e.g. reducing adult survival, adult fecundity, or juvenile survival). Under some forms of density regulation, a genotype that maximizes *r* will be uninvasible; while under others, a genotype that maximizes $R_{0}$ will be uninvasible. Again, model assumptions dictate the choice of fitness measure.

## A note on biological modelling

Takacs and Bourrat insist on the primacy of continuous-time over discrete-time growth models. We find this puzzling. While it is true that only a few species (such as 13-year periodical cicadas) correspond exactly to the assumptions of a discrete-time growth model with non-overlapping generations, such models can nonetheless yield insight; and of course, continuous-time models rest on idealizations of their own. There is no general reason to regard continuous-time models as “closer to biological reality” than discrete-time models, nor therefore to regard continuous-time fitness measures as more fundamental. The choice between modelling in discrete or continuous time, in both evolution and ecology, is a pragmatic matter; it is dictated partly by considerations of analytical or computational convenience—which can pull in either direction—and partly by the biology of the species under consideration.

It is worth noting that some mathematical phenomena only occur in a discrete-time setting. A famous example is the discrete logistic growth model discussed by May (1974). May shows that the discrete model displays a form of instability and oscillatory behaviour that is not present in the continuous model. So the discrete model (making use of difference equations) is, in a sense, more complex than the continuous model (making use of differential equations). Discrete modelling therefore has its rightful place in theoretical biology.

It should also be noted that some models with overlapping generations can be re-described as models with non-overlapping generations. Cannings (1973) shows how the continuous-time Moran model of genetic drift assuming overlapping generations can be reduced to a non-overlapping generations model by modifying its nomenclature. For this class of models, the choice between an overlapping and a non-overlapping generations assumption leaves the mathematics of the model essentially unaffected.

## The definition of fitness

Much ink has been spilled in both biology and philosophy of biology over the question of how to define fitness. It is tempting to assume, given the great generality of Darwin’s theory, that there must be a single “right” answer to this question. The tendency that Takacs and Bourrat criticize—taking the geometric mean number of offspring as “the” definition of fitness—is an example of this. But as we have seen, there are circumstances where this fitness measure (or its continuous-time analogue) is not suitable for answering certain questions of interest. The correct moral, though, is not to seek an alternative “always right” fitness measure, as Takacs and Bourrat do, but rather to realize that there is no universally correct measure. That is, the correct measure depends on both precisely what question we want the fitness measure to answer, and on model assumptions.

This leads to the question of what determines whether a candidate fitness measure is or is not “correct”, that is, what do we actually mean by “correct” here? Takacs and Bourrat say little about this, other than to talk about the measure being “predictively efficacious” or “predictively adequate” (2022, p.12). But the question is an important one, and has been extensively discussed in the biological literature (Mylius and Diekmann 1995; Metz et al. 1992; Saether and Engen 2015; Brommer 2000; Otto and Day 2007). The consensus that has emerged is that a fitness measure (or putative optimality criterion) should ideally be justified on the basis of an invasibility, or evolutionary stability, argument. That is, for a fitness measure to be “correct”, it should be the case that the type with the highest value of this measure is an ESS (and preferably the unique ESS) of the underlying evolutionary model. (The rationale here is that only an ESS is a candidate endpoint of the evolutionary process). This provides a principled way of choosing between fitness measures; and it picks out the geometric mean measure in those stochastic environment models where it is used. Moreover, it is this consideration that shows why the instantaneous rate of increase *r*—Takacs and Bourrat’s preferred measure—is not always the right fitness measure when there is density-dependence, even without the complication of fluctuating environments, as noted above.

The fact that there is no uniquely correct fitness measure, free from model assumptions, is perhaps somewhat surprising. It is tempting to think that there must be such a measure on pain of evolutionary theory lacking the generality that it is widely supposed to have. Surely, one is tempted to say, there must be some “meta-model” that subsumes all others and that gives a universally-applicable mathematical definition of fitness of which all others are special cases? But the evidence strongly suggests that there is no such definition; and evolutionary biology seems to get on fine without one. We suggest that this is because the theory of evolution is in reality closer to a “patchwork of models” (*sensu* Cartwright (2008)) than to an axiomatic theory, despite the impression conveyed by the simple verbal summaries of “the Darwinian principles” found in both the scientific and philosophical literature. Further philosophical reflection on this point might be useful.

## Footnotes

1 The presentation follows an example in Saether and Engen (2015).

2 Both *r* and $R_{0}$ can be expressed as functions of genotype-specific birth and death rates, however they are different functions. See Brommer (2000) for a good explanation of this point.
